# The Association of Proning and Stroke among Deeply Sedated Critically Ill SARS-CoV-2 (COVID-19) Patients

**DOI:** 10.1155/2022/6348888

**Published:** 2022-06-21

**Authors:** Keith Moon Q. Saberon, Jo-Ann Rosario Soliven

**Affiliations:** Department of Neurology, The Medical City, Ortigas Avenue, Pasig, Philippines

## Abstract

There has been an increasing incidence of stroke cases among SARS-CoV-2 (COVID-19) patients who were deeply sedated and underwent proning positioning. We reviewed the association of proning and sedations used to the development of stroke, including demographic profiles of patients with COVID-19 infection in the critical care unit. There was a significant association seen among COVID-19 patients in the ICU who underwent proning to the development of stroke, with up to 15 times risk of having stroke (*p* value = 0.007) than those who were not proned during their course of ICU stay. Patients who were given propofol and fentanyl as sedation during proning for more than 24 hours was significantly associated with the development of stroke (*p* value = 0.004). Patient risk factors were also studied (age variability, hypertension, diabetes, smoking, and alcoholism) and showed that patients who were alcoholic beverage drinkers were significantly associated to the development of stroke during proning (*p* value = <0.001). The usual risk factors for stroke in the general population (hypertension, diabetes, and cigarette smoking) were not associated with stroke development during proning, strengthening the fact that proning during COVID-19 infection is an independent risk factor for the development of stroke thus needing stroke surveillance during the duration of proning.

## 1. Introduction

There has been increasing incidence of stroke in the critically ill, deeply sedated proned SARS-CoV-2 (COVID-19)-infected patients. The hypercoagulability state of COVID-19-infected patients impacts the course of the disease. Although there has been no clear distinction between the number of comorbidities and the morbidity and mortality rates of these patients, there have been a significant number of patients who die of COVID-19 even without comorbidities. Part of the management of COVID-19 patients who developed acute respiratory distress syndrome (ARDS) is to do the proning position. Stroke is difficult to determine during the course of proning procedure since these patients are subjected to deep sedation, and there will be limited if not difficult neurologic assessment during the proning procedure. It is therefore unfortunate for postproned patients who were being weaned from sedation, examined to be unarousable, and diagnosed late to have stroke.

## 2. Methodology

### 2.1. Population and Sampling Method

All adult patients admitted to the critical care unit diagnosed with SARS-CoV-2 infection using RT-PCR from May 1, 2020, to May 31, 2021, were evaluated in this study. The sample size result was computed using OpenEpi, version 3, an open-source calculator. In order to get a 95% confidence level, a minimum population of 135 patients should be included in the study. The study evaluated a total of 206 patients which qualified the minimum amount required for the confidence level.

The study included a total of 206 patients which qualified inclusion criteria. Of these 206 patients, the 135 patients will be selected using the random sampling method. All patients who were admitted to the critical care unit from the specified time frame were included in the study. The decision for proning to a patient was made upon the clinical criteria of the critical care physician which includes a P/F ratio of less than 150 which signifies acute respiratory distress syndrome (ARDS).

### 2.2. Methods

All patients who were admitted due to COVID-19 infection in the critical care unit from May 1, 2020, until May 31, 2021, were reviewed using the MIDAS electronic system. Data acquired from the study subjects and laboratory values were tabulated and encoded. The study investigators were the only ones who were able to access the data gathered. To ensure data privacy, data obtained will be encoded in a password-protected Microsoft Excel file. Each study subject will be assigned number codes.

### 2.3. Statistical Analysis

Baseline characteristics such as demographics and comorbidities as well as study subject outcomes were gathered and tabulated. The odds ratios (OR) and their respective 95% confidence intervals (95% CI) for the binary outcomes of each study were presented. The chi-square test was used to test the association of sex, risk factors, and comorbidities to the occurrence of stroke. The binary logistic regression model was used to estimate the odds ratio of proning and sedation to the development of stroke. All valid data will be included in the analysis. Missing variables will be neither replaced nor estimated. The null hypothesis will be rejected at a 0.05*α*-level of significance.

### 2.4. Literature Review

Neurologic complications of SARS-CoV-2 or popularly known as COVID-19 infection are known and posed a grave threat to critically ill patients undergoing the acute phase of the disease. It is of great concern that during the critical phase of the disease, where patients develop acute respiratory distress syndrome and are placed on prone with deep sedation, the development of stroke is detected late.

Prone positioning (PP) of mechanically ventilated patients is an effective first-line intervention to treat patients with moderate to severe acute respiratory distress syndrome (ARDS) receiving invasive mechanical ventilation, as it improves gas exchanges and reduces mortality [1]. According to the Intensive Care Society, critical care clinicians treating these patients in critical care have reported that patients with moderate to severe ARDS appear to have responded well to invasive ventilation in the prone position, leading to prone ventilation being recommended in international guidelines for the management of COVID-19 [2].

Patients who were unable to awaken from deep sedation are common, and it is therefore late in the course to find that one of the causes of their unarousability is stroke. It is still unknown whether proning itself or the duration of the proning procedure and the effect of deep sedation can impact the incidence of stroke post proning. The duration of sedation can alter the course of the disease in these patients. A recent study by Brown et al. showed a significant relationship between prolonged sedation and the incidence of neurological complications in SARS-CoV-2-infected patients [3]. The study highlighted the effect of prolonged sedation and the increased chance of hypoxia which caused the neurological trauma.

There have been a number of complications caused by the infection of COVID-19. A study by Vinayagam and Sattu described the SARS-CoV-2 (COVID-19) infection as an entity potentially causing coagulability and thromboses and thus the risk for blood clots. It follows that the myriad of embolic events has the potential to send blood clots to any and all organs [4].

Complications during proning position vary from airway compromise to peripheral nerve injury, the significance of which is the development of arterial or venous thrombosis which can herald the development of cerebral infarction. Blood pressure is a significant parameter in which episodes of hypotension may promote cerebral ischemia causing watershed infarctions, or uncontrolled blood pressure spikes may damage the already injured blood vessel walls causing rupture and cerebral hemorrhage. The different complications during proning position were described by Rahmani et al. in an attempt to determine the contraindications of patients for proning [5]. They cited that the awake prone position combined with high-flow nasal cannula (HFNC) therapy could be applied safely and efficiently in severe COVID-19 patients as well as it may lessen the conversion to critical illness and the requirement for tracheal intubation.

Interest in propofol as a sedative agent is increasing. Sedation by continuous infusion of midazolam in ICU patients may lead to accumulation and prolonged recovery [6]. A study by Beller et al. showed that sedation with propofol provided rapid control of the level of sedation and when infusion was discontinued, there was adequate recovery and decrease in blood propofol concentration were similar after 24, 48, 72, and 96 h of infusion. Cumulative effects, tachyphylaxis, or other untoward effects were also not observed [6].

All of these factors, the duration of sedation and proning, complications during proning, and the hypercoagulable state of the SARS-CoV-2 infection itself render the critically ill patient a high risk for the development of stroke that can develop during the critical phase of the illness. Therefore, the diagnosis of stroke is hindered by the deep sedation and the lack of neurological assessment during the time of proning.

## 3. Results

A total of 206 patients were enrolled in the trial from May 1, 2020, to May 31, 2021, and all patients with a positive COVID-19 RT-PCR admitted to the critical care unit were included in the study. Baseline characteristics are shown in Table 1. Out of the 135 patients in the study, 32 patients developed acute respiratory distress syndrome (ARDS) and underwent proning position. The baseline characteristics were generally balanced between the two groups except those patients who underwent proning have slight predominance of having hypertension. The percentage of patients who were smokers was slightly higher in the nonproned group as compared to the group who underwent proning. A total of 103 patients who tested COVID-19-positive and underwent treatment did not undergo proning.

The study showed that gender was not significantly associated with the development of stroke (*p* value = 0.822). Those patients with hypertension (*p* value = 0.967), diabetes (*p* value = 0.849), and who were cigarette smokers (*p* value = 0.453) were also not associated with stroke in either prone or unproned patients. However, alcohol intake has a significant association with the development of stroke (*p* value = <0.001) in which all patients who developed stroke were cigarette smokers (Table 2).

The mean age of patients who developed stroke was 64 years old (SD = 15.172) and for those that did not develop stroke was 65.33 (SD = 15.104). Age itself is significantly associated with the development of stroke (*p* value = 0.022, Table 3). Patients who underwent proning developed stroke (40.6%) with infarct (25%) as the most common type, followed by ICH (12.5%) and subarachnoid hemorrhage (3.1%). No patient who was proned developed stroke (Table 4).

There were a total of 32 severe COVID-19 patients with ARDS who underwent proning and 15 (14%) patients who had severe COVID-19 infection and ARDS. There is a 40.6% stroke rate among those who were proned as compared to those who were not proned (Table 5) in which all unproned patients did not develop stroke (0%).

All patients who underwent proning developed stroke, showing a statistically significant effect of its association to the development of stroke (*p* value = 0.007, Table 6). This showed almost 15 times risk of developing stroke among proned patients. All patients who developed stroke during proning used propofol as sedation, making its use having huge significant effect to stroke development during proning. Fentanyl use as sedation during proning also has a significant effect to stroke development (*p* value = 0.004, Table 7).

The duration of sedation was grouped into 2 variables: those whose sedation lasted only less than or equal to 24 hours, and those whose sedation lasted more than 24 hours. Duration of sedation and proning to more than 24 hours showed a significant effect on the development of stroke (*p* value= < 0.001).

## 4. Discussion

The results of our study showed that sex, comorbidities, and risk factors of critically ill SARS-CoV-2-infected patients in the ICU did not show a significant association to the development of stroke. There are patients who developed critical COVID-19 infection and stroke even without comorbid conditions. Few published studies explained the mechanism of stroke in COVID-19, and the infection per se has a 5% risk of developing stroke; however, most of these studies did not attribute proning and sedation as one of the contributing factors to its development [7]. Patients who were known to be hypertensive and diabetic versus those who were not may have an equal risk of developing stroke once they have been critically ill with COVID-19 infection. Cigarette smoking has been an established risk for the development of stroke; however, patients in this study who were chronic cigarette smokers and alcoholic beverage drinkers have not shown significant association with the development of stroke during COVID-19 critical illness. All these data strengthen the fact that the mechanisms of stroke in COVID-19 infection are based on inflammation-induced endothelial injury, hypercoagulability, and microvascular thrombosis during cytokine storm which was indicated in the article by Spence et al. and may not be related to the stroke caused by atherothrombosis seen on smokers, hypertensive, and diabetic patients. Another factor is sex, in which neither male nor female sex, did not show any significant relationship to the development of stroke.

Our study highlighted the high rate of cerebral infarction among proned (*N* = 8, 25%) in contrast to unproned patients (*N* = 0, 0%) during the critical stage of COVID-19 illness compared to those with hemorrhagic type of stroke. The hypercoagulable state during the acute stage of COVID-19 critical illness may explain the higher rate of infarcts versus hemorrhage. Although, factors such as the duration and type of sedation used during proning position may have an effect on its development. A comparison between patients with severe COVID-19 infection with ARDS who are proned versus not proned showed an alarmingly high rate of stroke among patients who were proned, in contrast to those unproned which has no stroke occurrence. This might indicate that proning itself may be an independent risk factor for stroke among patients with severe COVID-19 infection with ARDS.

In this study, there was a preference for the use of propofol due to its immediate sedative effect and fewer sedative-related complications. In this study, the maximum duration of propofol use was 48 hours to prevent the so-called propofol infusion syndrome. Nevertheless, its use as sedation is linked to the high stroke rate post proning in these critically ill COVID-19 patients. There still is no clear mechanism for the association of propofol and the development of stroke in these patients; however, the hypotensive effect, hypertriglyceridemia, and the arrhythmogenic effect of propofol may somehow link its effect to the development of stroke. This finding raises concern among critically ill patients since several studies pertaining stroke and COVID-19 infection showed that the proportion of patients with hypertension, diabetes, hyperlipidemia, atrial fibrillation, and congestive heart failure was significantly higher among those with acute ischemic stroke [8], and the duration of sedative use was not included as a parameter.

Patients who underwent proning showed a huge significant relationship to the development of stroke wherein all who developed stroke were proned under the sedation of propofol and fentanyl, while those who were sedated with Precedex and midazolam did not show any statistical significance. Midazolam has been widely used among critical care units especially for sedation during mechanical ventilation due to its potency, rapid onset, and short duration of action [9]. The duration of patients who were proned was divided into less than or equal to 24 hours, and those patients proned for more than 24 hours developed stroke, making it statistically associated with the development of stroke in proned patients (*p* value < 0.001). This indicates that proning more than 24 hours increases the risk of stroke by 12 times as compared to those who are not proned.

A study by Joswiak et al. indicated the benefit of prone position among patients diagnosed with ARDS by increasing the ratio of arterial oxygen partial pressure over inspired oxygen fraction and the right and left cardiac preload [10]; however, there has been no study that links the mechanism of stroke during the prone position to changes in cardiac hemodynamics, especially in a critically ill SARS-CoV-2 virus-infected patient.

## 5. Conclusion

Critically ill patients diagnosed with SARS-CoV-2 (COVID-19) infection who underwent proning are associated with an increased risk of developing stroke with infarction as the predominant etiology. The use of propofol and fentanyl as sedation during proning for more than 24 hours also is associated with an increased risk of developing stroke.

## Figures and Tables

**Table 1:** Baseline characteristics of patients.

	Proned (*N* = 32)	Not proned (*N* = 103)
Age ≥60 yr (no. (%))	21 (65)	67 (65)
Male sex (no. (%))	12 (37)	31 (30)
Hypertension	26 (81)	72 (70)
Diabetes	12 (37)	38 (36)
Smoking	12 (37)	48 (46)
Alcohol intake	16 (50)	56 (54)
Developed stroke	13 (40)	0 (0)

**Table 2:** Clinical predictors of stroke.

Clinical predictors		Stroke		*p* value
		No	Yes	
Sex	Female	38	5	0.822
	Male	84	8	

Hypertension	No	34	3	0.967
	Yes	88	10	

Diabetes	No	76	9	0.849
	Yes	46	4	

Smoking	No	66	9	0.453
	Yes	56	4	

Alcohol intake	No	56	7	<0.001
	Yes	122	13	

**Table 3:** Association of age and stroke.

	Stroke		*p* value (Mann–Whitney *U*)
	No	Yes	
Age (mean)	65.33 (15.104)	64.39 (15.172)	0.022

**Table 4:** Stroke rate in proned vs not proned.

	Infarct	ICH	SAH	Total
Proned	8 (25%)	4 (12.5%)	1 (3.1%)	13 (40.6%)
Not proned	0 (0%)	0 (0%)	0 (0%)	0 (0%)

**Table 5:** Stroke rate between severe COVID-19 patients with ARDS who were proned vs not proned.

Severe COVID-19 infection with ARDS	With stroke	No stroke
Proned (*N* **=** 32)	13 (40.6%)	19 (59.3%)
Not proned (*N* **=** 15)	0 (0)	15 (100%)

**Table 6:** Proning and occurrence of stroke.

Variables in the equation									
		*B*	S.E.	Wald	d*f*	*p* value	Odds ratio	95% CI for odds ratio	
								Lower	Upper
Step 1a	Proning	1.562	0.582	7.203	1	0.007	4.769	1.524	14.920
	Constant	−3.248	0.510	40.631	1	0.000	0.039		

**Table 7:** Sedations used and occurrence of stroke.

Variables in the equation								
	*B*	S.E.	Wald	d*f*	*p* value	Odds ratio	95% CI for odds ratio	
							Lower	Upper
Propofol	20.436	4145.588	0.000	1	0.996	7500042087.086	0.000	
Fentanyl	1.845	0.635	8.437	1	0.004	6.328	1.822	21.976

## Data Availability

The data used to support this study are taken from previously published articles and are cited as references.
